# Meropenem Administered via Intravenous Regional Limb Perfusion for Orthopedic Sepsis in Horses: A Clinical Retrospective Study

**DOI:** 10.3389/fvets.2021.629627

**Published:** 2021-03-26

**Authors:** Allison P. Mosichuk, Joseph S. Smith, Dane M. Tatarniuk, Jarrod R. Troy, Amanda J. Kreuder

**Affiliations:** ^1^College of Veterinary Medicine, Iowa State University, Ames, IA, United States; ^2^Veterinary Diagnostic and Production Animal Medicine, College of Veterinary Medicine, Iowa State University, Ames, IA, United States; ^3^Biomedical Sciences, College of Veterinary Medicine, Iowa State University, Ames, IA, United States; ^4^Veterinary Clinical Sciences, College of Veterinary Medicine, Iowa State University, Ames, IA, United States; ^5^Veterinary Microbiology and Preventive Medicine, College of Veterinary Medicine, Iowa State University, Ames, IA, United States

**Keywords:** antibiotics, carbapenem, equine, gentamicin, meropenem, septic synovitis, regional limb perfusion

## Abstract

Septic synovitis is a critical orthopedic condition in horses. Early intervention is key, with antibiotic therapy typically initiated prior to culture and susceptibility reports becoming available. The pharmacokinetics of several antibiotics have been studied in horses for use in intravenous regional limb perfusion (IVRLP) for septic synovitis, including the carbapenem antibiotic, meropenem. For a variety of factors, some veterinary clinicians may select IVRLP meropenem as therapy for these cases. Meropenem is a vital antibiotic in human medicine, making veterinary use divisive. However, verifying the efficacy of meropenem contrasted to other IVRLP antibiotics is essential for appropriate antimicrobial stewardship. To investigate this, equine patient medical records at a single veterinary teaching hospital were examined. Cases treated with meropenem or gentamicin via IVRLP for septic synovitis were retrospectively analyzed for demographics, diagnostics, treatments, outcomes, and adverse effects. Twenty-three meropenem and 37 gentamicin treated horses were analyzed; demographic information was similar between groups. In the meropenem group, nine horses received meropenem only; the remainder received another antibiotic initially then changed to meropenem. Structures infected included joints (meropenem = 13, gentamicin = 17), tendon sheaths (meropenem = 5, gentamicin = 8) and navicular bursae (meropenem = 2, gentamicin = 6). Overall survival to discharge was 86% (52/60), with meropenem 91% (21/23) and gentamicin 84% (31/37), with no statistically significant differences noted between meropenem or gentamicin groups for overall survival to discharge or outcome after discharge. Twenty-four of 26 bacterial isolates obtained from culture were reported as sensitive to imipenem, a carbapenem antibiotic similar to meropenem. Reported susceptibility to other antibiotics such as ceftiofur (*n* = 22/26), ampicillin (*n* = 18/26), amikacin (*n* = 15/26), or gentamicin (*n* = 12/26) was also frequently present. In the population of this study, antimicrobial activity augmented with IVRLP using either meropenem or gentamicin both appear to be an effective treatment for septic synovial structures, therefore, less critical antimicrobials may be a viable and more judicious treatment option.

## Introduction

Meropenem, a carbapenem antibiotic, is included in the beta-lactam family of antibiotics. The mechanism of action of beta-lactam antibiotics is to inhibit cell wall synthesis leading to cell lysis in a time dependent manner. Carbapenem antibiotics are effective against gram positive or negative bacteria, however, when used parenterally, they require frequent (multiple administrations per day) dosing due to a short half-life (1–4). According to the World Health Organization (WHO), meropenem is a critically important antibiotic in human medicine for multi-drug resistant (MDR) infections (5). Thus, it has been recommended to be used sparingly in veterinary medicine to avoid selecting for antimicrobial resistant organisms (5, 6). Many veterinary institutions, including the institution represented in this study, have protocols in place which restrict the use of carbapenems to the rare use for severe, life threatening conditions in individual animals when culture and susceptibility testing indicates that it is the only suitable option.

Septic synovitis is an orthopedic emergency with high morbidity, with discharge to hospital survival ranging from 38.5 to 86% (7, 8). Early, aggressive treatment with lavage and antibiotics has been associated with higher survival rates (7). Multi-drug resistant or gram-negative synovial infections have been associated with reduced survival (8). Intravenous regional limb perfusions (IVRLP), a common procedure used for treating septic synovial structures in horses, allow for higher concentrations of antibiotics targeting the affected structures while decreasing the adverse effects of systemic antibiotic treatment (9). Additionally, IVRLP may increase survival rates when used in conjunction with other septic synovitis therapy (10). For distal limb perfusions in horses a tourniquet or pressure cuff is placed proximal to the affected joint for approximately 15–30 min and a catheter is placed into the palmar/plantar digital vein to administer the selected antibiotic (9). The pharmacokinetic parameters of multiple drugs, including amikacin, penicillin, gentamicin, vancomycin, enrofloxacin, chloramphenicol, ceftiofur, imipenem and meropenem have been evaluated for IVRLP use in horses (11, 11–19). The most common bacteria associated with equine septic synovitis have been shown to have susceptibility to antibiotics other than meropenem (7, 8, 20–22). However, due to the perceived risk of MDR infection some veterinarians use meropenem in contrast to WHO guidelines.

If a critically important antibiotic, like meropenem, is to be used in veterinary species, it is imperative to evaluate its efficacy and clinical outcomes (6). There is concern meropenem use in IVRLP could lead to subtherapeutic concentration and increased antimicrobial resistance to this vital antibiotic (11, 23). Human patients treated parenterally with meropenem may experience adverse effects such as nausea, diarrhea, pain at injection site, seizures, and changes in hepatic biochemistry (19, 24). IVRLP in humans and other species has been associated with adverse effects such as thrombosis, venous scarring, and bruising, however, previous studies have found no adverse effects when used for IVRLP in healthy equine patients, (25–27). As such, IVRLP presents the chance to reduce adverse effects by significantly less drug exposure than by parental administration. The primary objective of this retrospective study of clinical cases was to compare clinical parameters, short term survival, and long term survival in equine orthopedic sepsis cases treated with antimicrobial therapy augmented with IVRLP using either meropenem or gentamicin.

## Materials and Methods

Medical records were obtained from the Lloyd Veterinary Medical Center at Iowa State University's College of Veterinary Medicine. Records were screened for use of meropenem or gentamicin via IVRLP for orthopedic sepsis in equine patients treated in hospital from December 1, 2016 to March 1, 2020. Orthopedic sepsis was defined as an infection of an orthopedic structure as diagnosed by at least one of the following being consistent with infection: bacterial culture, cytology (28), diagnostic imaging results consistent with infection. Horses that received IVRLP with both meropenem and gentamicin were excluded from the gentamicin group. Any cases that used meropenem via a different route of administration, such as systemically or intra-articular, were excluded from the study. Information recorded included clinical parameters (age, breed, weight, sex, and body condition score), synovial structure involved, reported cause for synovial sepsis (hematogenous, intra-synovial injection, penetrating wound), IVRLP technique, previous/current systemic medications, surgical (arthroscopic lavage under general anesthesia) vs. non-surgical lavage, diagnostics performed, adverse effects, and survival to hospital discharge.

Diagnostic reports for cytology, culture and susceptibility, blood work, and various imaging methods were recorded. From cytology data, group cell count and protein concentrations were compared for normality, and then with an appropriate parametric or non-parametric test via commercial statistical software (GraphPad Prism 8.0, GraphPad Software, San Diego, CA) with a *p* < 0.05 considered statistically significant.

Owners of cases that survived to hospital discharge were contacted by phone and or email, from June 6 to July 10, 2020 to answer a script of standardized follow-up questions regarding survival outcome data (Supplementary File 1). Clients were asked what the patient's activity level was before hospitalization, if they returned to a prior level of activity after discharge, if they had additional treatments, and if the patient experienced any adverse effects. Outcome information between groups was compared with a commercial software program (GraphPad Prism 8.0, GraphPad Software, San Diego, CA) using Fisher's exact test with a *p* < 0.05 considered statistically significant.

## Results

### Signalment

The signalment demographics for both meropenem and gentamicin IVRLP groups are displayed in Table 1. The signalment, including age, sex, and breed, is similar between the meropenem and gentamicin groups.

**Table 1:** Demographic information for horses administered either meropenem or gentamicin via intravenous regional limb perfusion (IVRLP).

**Group**	**Total Number**	**Female (*n)***	**Intact Male (*n*)**	**Castrated Male (*n)***	**Age (years, avg +/– SD)**	**Breeds (*n)***	**Weight (kg, avg +/– SD)**
Meropenem IVRLP	23	11	1	11	7.46 +/− 5.50	Quarterhorse (9) Paint (3) Missouri Fox Trotter (2) Standardbred (2) Thoroughbred (2) Appaloosa (1) Arabian (1) Hackney (1) Hafflinger (1) Hanoverian (1)	460 +/− 153
Gentamicin IVRLP	37	26	4	7	8.23 +/− 6.14	Quarterhorse (21) Percheron (4) Thoroughbred (4) Paint (3) Standardbred (1) Mustang (1) Shetland Pony (1) Warmblood (1) Belgian (1)	484 +/− 228

### Diagnosis

#### Meropenem

The diagnoses in the meropenem group are displayed in Table 2. These diagnoses were due to trauma (*n* = 9), iatrogenic causes (*n* = 8), unknown reasons (*n* = 4), or presumptive hematogenous bacterial spread (*n* = 2). These conditions affected the horses' left hind (*n* = 13), right hind (*n* = 6), left front (*n* = 2), and right front (*n* = 2) limbs. Of the 15 horses that had infected joints, the metatarsal/metacarpal-phalangeal joint (*n* = 5), the distal interphalangeal joint (*n* = 4), the proximal interphalangeal joint (*n* = 3), and the tarsocrural joint (*n* = 3) were affected. Nineteen horses were hospitalized for their diagnosis once, but others were admitted for a second (*n* = 3) and third (*n* = 1) hospitalization due to re-occurrence of infection.

**Table 2:** Diagnoses for horses administered either meropenem or gentamicin via intravenous regional limb perfusion (IVRLP).

**Group**	**Diagnosis**	***n***
Meropenem (*n* = 23)	Septic arthritis	12
	Septic tenosynovitis	5
	Septic navicular bursa	2
	Septic arthritis and tenosynovitis	2
	Septic physitis and arthritis	2
Gentamicin (*n* = 37)	Septic arthritis	17
	Septic tenosynovitis	8
	Septic navicular bursa	6
	Septic arthritis and tenosynovitis	3
	Septic physitis and arthritis	1
	Septic calcanean bursa	1
	Septic arthritis and navicular bursa	1

#### Gentamicin

The diagnoses of the 37 equine patients receiving gentamicin IVRLP are displayed in Table 2. These diagnoses were due to trauma (*n* = 21), unknown reasons (*n* = 9), iatrogenic causes (*n* = 4), or presumptive hematogenous bacterial spread (*n* = 3). These conditions affected the horses' left hind (*n* = 14), right hind (*n* = 9), left front (*n* = 7), right front (*n* = 5) limbs, or more than one limb (*n* = 2). Of the 22 horses that had infected joints, the distal interphalangeal joint (*n* = 9), the metatarsal/metacarpal-phalangeal joint (*n* = 4), multiple joints (*n* = 4), the proximal interphalangeal joint (*n* = 3), or the tarsocrural joint (*n* = 2) were affected. Thirty-five of the horses were hospitalized for their diagnosis once, but others were admitted for a second (*n* = 2) hospitalization.

### Diagnostics

#### Meropenem

Diagnostic procedures performed in the meropenem group include: radiographs (*n* = 20), culture and susceptibility (*n* = 15), ultrasound (*n* = 14), cytology (*n* = 10), computed tomography (CT, *n* = 1), magnetic resonance imaging (MRI, *n* = 1), and nuclear scintigraphy *(n* = 1). Radiographic reports revealed soft tissues swelling (*n* = 9), normal anatomy (*n* = 5), changes to osseous structures (*n* = 4), and penetrating nails (*n* = 2). Ultrasound reports were not provided in the records. CT was used to diagnose inflammatory/septic tenosynovitis of the left tarsal sheath in one equine patient. MRI was used to diagnose moderate collateral sesamoidean ligament desmopathy and moderate distal interphalangeal joint effusion in another equine patient in the study.

Ten of the 23 horses in the meropenem group had synovial fluid evaluated by cytology, with fourteen samples total. Structures sampled included joint fluid (*n* = 11) and tendon sheath fluid (*n* = 3). Cytology data for the meropenem group is displayed in Table 3. Fifteen horses had joint or synovial fluid submitted for culture, with a total of 16 samples. Of the 15 horses with cultures, eight cases received culture results before starting meropenem whereas seven received the results after already starting meropenem. The susceptibility data showed that out of 16 bacterial isolates with sensitivities reported, 15 were reported as sensitive to imipenem, 13 to ceftiofur, 11 to ampicillin, eight to amikacin, and five to gentamicin. All 15 of the isolates sensitive to imipenem were also sensitive to other antibiotics. Culture and susceptibility results for horses treated with meropenem IVRLP are included by case in Table 4.

**Table 3:** Synovial fluid characteristics for groups of horses receiving meropenem (upper) and gentamicin (lower) via intravenous regional limb perfusion (IVRLP).

	***n***	**Average ± SD**	**Minimum**	**Maximum**
**Meropenem**				
Cell count (/μl)	14	58,487 ± 67,035^a^	640	203,310
Protein (gm/dl)	13	5.42 ± 1.43^b^	2.40	7.70
pH	14	6.51 ± 0.24^c^	6.00	6.80
**Gentamicin**				
Cell count (/μl)	15	42,747 ± 70,424^a^	450	260,170
Protein (gm/dl)	15	4.35 ± 1.75^b^	2.20	7.00
pH	15	6.55 ± 0.21^c^	6.40	7.00

a *Comparisons between the groups for cell count were not statistically significant (P = 0.2375)*.

b *Comparisons between the groups for protein concentration were statistically significant (P = 0.0371)*.

c *Comparisons between the groups for pH were not statistically significant (P = 0.8131)*.

**Table 4:** Bacterial culture and susceptibility results of horses treated with Meropenem *via* intravenous regional limb perfusion with growth characteristics as reported by the laboratory.

					**Susceptibility results (MIC μg/ml)**					
					**Concentration-dependent**		**Time-dependent**			
**Case #**	**Diagnosis**	**Culture source**	**Bacterial species isolated**	**Growth**	**Amikacin**	**Gentamicin**	**Ampicillin**	**Ceftiofur**	**Imipenem**	**Survival to discharge**
1	Septic tenosynovitis	Joint fluid	*Streptococcus zooepidemicus*	Couple	>32 / R	>8 / R	≤0.25 / S	≤0.25 / S	≤1 / S	Yes
2	Septic arthritis	Joint material	*Truperella pyogenes*	Few	≤4 / S	≤1 / S	≤0.25 / S	≤0.25 / S	≤1 / S	Yes
			*Streptococcus dysgalactiae ss equisimilis*	Enrichment only	>32 / R	4 / S	≤0.25 / S	≤0.25 / S	≤1 / S	
			*Clostridium sporogenes*	Low	>32 / R	>8 / R	≤0.25 / S	>4 / R	≤1 / S	
3	Septic tenosynovitis	Joint swab	*Streptococcus zooepidemicus*	Positive	>32 / R	>8 / R	≤0.25 / S	≤0.25 / S	≤1 / S	Yes
			*Enterococcus faecalis*	Couple	>32 / R	>8 / R	1 / S	>4 / R	≤1 / S	
4	Septic arthritis	Joint fluid	*Staphylococcus species*	Positive	NP	NP	NP	NP	NP	No
5	Septic arthritis	Joint swab	*Escherichia coli* (haemolytic)	Couple	≤4 / S	>8 / R	>32 / R	0.5 / S	≤1 / S	Yes
6	Septic physitis and arthritis	Joint swab, fluid and material	No growth		NP	NP	NP	NP	NP	Yes
7	Septic arthritis	Joint fluid	*Staphylococcus aureus*	Low	≤4 / S	>8 / R	0.5 / R	1 / S	≤1 / S	No
8	Septic arthritis	Joint fluid	*Streptococcus zooepidemicus*	Heavy	>32 / R	>8 / R	≤0.25 / S	≤0.25 / S	≤1 / S	Yes
9	Septic arthritis	Joint fluid	No growth		NP	NP	NP	NP	NP	Yes
		Tendon fluid	*Bacillus licheniformis*	Positive	NP	NP	NP	NP	NP	
10	Septic arthritis and tenosynovitis	Joint swab	*Staphylococcus aureus*	Low	≤4 / S	≤1 / S	≤0.25 / S	1 / S	≤1 / S	Yes
11	Septic arthritis	Joint fluid	No growth		NP	NP	NP	NP	NP	Yes
12	Septic arthritis and tenosynovitis	Joint fluid	No growth		NP	NP	NP	NP	NP	Yes
13	Septic arthritis	Joint fluid	*Staphylococcus aureus*	Heavy pure (enrichment only)	≤4 / S	>8 / R	1 / R	1 / S	≤1 / S	Yes
14	Septic arthritis	Abscess, swab	*Streptococcus zooepidemicus*	Heavy	>32 / I	8 / I	≤0.25 / S	≤0.25 / S	≤1 / S	Yes
15A	Septic tenosynovitis	Draining lesion	*Actinobacillus equuli*	Few	8 / S	4 / I	≤0.25 / NI	≤0.25 / S	≤1 / S	Yes
15B	Septic tenosynovitis	Abscess, swab	*Enterococcus faecalis*	Heavy mixed	>32 / R	>8 / R	1 / S	>4 / R	2 / I	
			*Streptococcus zooepidemicus*	Heavy mixed	16 / S	4 / S	≤0.25 / S	≤0.25 / S	≤1 / S	
			*Proteus* sp.	Heavy mixed	≤4 / S	≤1 / S	>32 / R	≤0.25 / S	≤1 / S	

*S, susceptible; I, intermediate; R, resistant; NI, no interpretation because guidelines have not been established by the Clinical and Laboratory Standards Institute (CLSI) for the bacteria/drug combination being tested. NP: susceptibility not performed. Susceptibility results are displayed in concentration of micrograms/milliliter. Case 15 had two separate cultures performed*.

#### Gentamicin

Diagnostic procedures performed in the gentamicin group include: radiographs (*n* = 31), culture and susceptibility (*n* = 13), ultrasound (*n* = 13), cytology (*n* = 12), and MRI (*n* = 1). Radiographic reports revealed soft tissues swelling (*n* = 17), penetrating wounds (*n* = 6), changes to boney structures (*n* = 6), and normal anatomy (*n* = 2). Ultrasound reports were not provided in the records for the gentamicin group. MRI was used to diagnose a laceration of the soft tissues proximolateral to the left metatarsophalangeal joint in one equine patient in the study.

Twelve of the 37 gentamicin group horses had synovial fluid evaluated by cytology, with seventeen samples total. Structures sampled included joint fluid (*n* = 10) and tendon sheath fluid (*n* = 7). Cytology data for the gentamicin group is displayed in Table 3. The susceptibility data showed that, out of ten bacterial isolates with sensitivities reported, nine were reported by the laboratory as sensitive to imipenem, nine to ceftiofur, seven to amikacin, seven to gentamicin, and seven to ampicillin. All nine of the isolates sensitive to imipenem were also sensitive to other antibiotics. Culture and susceptibility results for horses treated with gentamicin IVRLP are included by case in Table 5.

**Table 5:** Bacterial culture and susceptibility results of horses treated with Gentamicin via intravenous regional limb perfusion with growth characteristics as reported by the laboratory.

					**Susceptibility results (MIC μg/ml)**					
					**Concentration-dependent**		**Time-dependent**			
**Case #**	**Diagnosis**	**Culture source**	**Bacterial species isolated**	**Growth**	**Amikacin**	**Gentamicin**	**Ampicillin**	**Ceftiofur**	**Imipenem**	**Survival to discharge**
1	Septic physitis and arthritis	Joint fluid	No growth		NP	NP	NP	NP	NP	No
2	Septic tenosynovitis	Joint fluid	No growth		NP	NP	NP	NP	NP	Yes
3	Septic arthritis	Joint fluid	No growth		NP	NP	NP	NP	NP	Yes
4	Septic tenosynovitis	Tendon Sheath Fluid	*Streptococcus zooepidemicus*	Heavy	>32 / R	>8 / R	≤0.25 / S	≤0.25 / S	≤1 / S	Yes
			*Eschericha coli*	Heavy	≤4 / S	≤1 / S	4 / S	0.5 / S	≤1 / S	
			*Streptococcus uberis*	Moderate	NP	NP	NP	NP	NP	
			*Proteus vulgaris*	Heavy	≤4 / S	≤1 / S	>32 / R	0.5 / S	≤1 / S	
5	Septic tenosynovitis	Joint fluid	Staphylococcus epidermidis^*^	Positive	≤4 / S	>8 / R	4 / R	>4 / R	≤1 / R	Yes
6	Septic arthritis	Joint fluid	*Streptococcus dysgalactiae ss. equisimilis*	Heavy	32 / I	4 / S	≤0.25 / S	≤0.25 / S	≤1 / S	Yes
			*Escherichia coli*	Heavy	≤4 / S	≤1 / S	4 / S	0.5 / S	≤1 / S	
7	Septic arthritis and navicular bursa	Tendon	*Paenibacillus amylolyticus*	Positive	NP	NP	NP	NP	NP	No
8	Septic arthritis	Tendon	No growth		NP	NP	NP	NP	NP	Yes
9	Septic arthritis	Joint fluid	*Salmonella* species group B	Moderate	≤4 / S	≤1 / S	1 / S	1 / S	≤1 / S	Yes
10	Septic tenosynovitis	Joint fluid	*Streptococcus dysgalactiae ss. equisimilis*	Heavy	32 / I	8 / I	≤0.25 / S	≤0.25 / S	≤1 / S	Yes
11	Septic arthritis and tenosynovitis	Joint fluid	*Serratia marcescens*	Few	≤4 / S	≤1 / S	>32 / R	1 / S	≤1 / S	No
12	Septic navicular bursa	Joint fluid	*Escherichia coli*	Positive	≤4 / S	≤1 / S	2 / S	0.5 / S	≤1 / S	Yes
13	Septic arthritis and tenosynovitis	Joint fluid	No growth		NP	NP	NP	NP	NP	Yes

*S, susceptible; I, intermediate; R, resistant; NI, no interpretation because guidelines have not been established by the Clinical and Laboratory Standards Institute (CLSI) for the bacteria/drug combination being tested. NP: susceptibility not performed. Susceptibility results are displayed in concentration of micrograms/milliliter*.

* *The isolate was oxacillin resistant and PBP2A positive; indicating the isolate carries the mecA gene*.

#### Comparisons

Comparisons between groups for cell count of fluid submitted for cytology revealed no significant differences (meropenem: 58,487 ± 67,035 cells/μl; gentamicin: 42,747 ± 70,424; *p* = 0.2375). Comparisons of protein concentration revealed a significant difference (meropenem: 5.42 ± 1.43 gm/dL; gentamicin: 4.35 ± 1.75; *p* = 0.0371). Comparisons of joint fluid pH revealed no significant differences (meropenem: 6.51 ± 0.24; gentamicin: 6.55 ± 0.21; *p* = 0.8131).

### Treatment Regimen

#### Meropenem

Each of the 23 horses in the meropenem group was treated with at least one IVRLP of the affected limb, with the median number of IVRLP with meropenem being 2 (range 1–7). Horses received IVRLP with other medications, such as gentamicin (*n* = 12), amikacin (*n* = 1), or both gentamicin and amikacin (*n* = 2) before switching to meropenem. There were also nine horses that received meropenem as the first drug given via IVRLP. Of the 15 horses that switched to meropenem, the reasons for the change in therapy included: showing no improvement with other treatments (*n* = 7), not specifically stated (*n* = 5), or culture and susceptibility results (*n* = 3).

All of the meropenem group IVRLP were performed using 1 gram (one vial) of meropenem, combined with saline and/or mepivacaine. Dilution and application information is present in Supplementary File 2.

Of the 23 horses in the meropenem group, all of them received systemic antibiotics as well as anti-inflammatories. Concurrently administered antibiotics for the meropenem IVRLP group is displayed in Table 6. The concurrently administered anti-inflammatories for the meropenem IVRLP group are displayed in Table 7. Surgery reports were also evaluated for the 16 cases that underwent a surgical procedure while hospitalized. These procedures included through-and-through needle lavage only (*n* = 5), arthroscopy/tenoscopy and lavage (*n* = 6), lavage and debridement (*n* = 4), and arthrodesis (*n* = 1). All 16 of these procedures were done under general anesthesia.

**Table 6:** Concurrently administered antimicrobials for horses administered either meropenem or gentamicin via intravenous regional limb perfusion (IVRLP).

**Group**	**Antibiotic**	***n***
Meropenem (*n* = 23)	Gentamicin	19
	Trimethoprim/sulfadiazine	14
	Procaine penicillin G	10
	Amikacin	4
	Ceftiofur sodium	4
	Chloramphenicol	4
	Ceftiofur crystalline free acid	3
	Enrofloxacin	2
	Metronidazole	2
	Doxycycline	1
	Rifampin	1
Gentamicin (*n* = 37)	Gentamicin	25
	Trimethoprim/sulfadiazine	23
	Procaine penicillin G	18
	Ceftiofur crystalline free acid	5
	Amikacin	4
	Ceftiofur sodium	3
	Chloramphenicol	2
	Metronidazole	2
	Clarithromycin	1
	Enrofloxacin	1
	Rifampin	1

**Table 7:** Concurrently administered anti-inflammatories for horses administered either meropenem or gentamicin via intravenous regional limb perfusion (IVRLP).

**Group**	**Anti-inflammatory**	***n***
Meropenem (*n* = 23)	Phenylbutazone	21
	Flunixin meglumine	4
	Dexamethasone	1
	Diclofenac	1
Gentamicin (*n* = 37)	Phenylbutazone	31
	Flunixin meglumine	7
	Firocoxib	1

#### Gentamicin

Of the IVRLP performed using gentamicin, all received at least one IVRLP with gentamicin, with a median number of gentamicin treatments being 3 (range 1 to 11). All were combined with saline and/or mepivacaine. Dilution and application information is present in Supplementary File 2.

Of the 37 horses in the gentamicin group, all 37 of them received systemic antibiotics as well as anti-inflammatories. Concurrently administered antibiotics for the gentamicin IVRLP group is displayed in Table 6. The concurrently administered anti-inflammatories for the gentamicin IVRLP group is displayed in Table 7. Surgery reports were also evaluated for the 19 cases that underwent a surgical procedure while hospitalized. These procedures included through-and-through needle lavage only (*n* = 10), arthroscopy/tenoscopy only (*n* = 3), navicular bursotomy (*n* = 3), sequestrum removal and through-and-through needle lavage (*n* = 2), and through-and-through needle lavage and wound debridement (*n* = 1). All but one of these procedures were done under general anesthesia, with one exception, which was performed with an abaxial distal limb block.

### Outcomes

#### Meropenem

Twenty-one of the 23 horses (91%) in the meropenem group survived to discharge, but two were euthanized in hospital due to poor response to intervention. The two horses that were euthanized prior to discharge were diagnosed with septic arthritis of the distal interphalangeal joint and septic arthritis of the proximal interphalangeal joint. These horses were treated for 12 and 29 days respectively. Adverse effects due to some aspect of the treatment regimen or hospitalization was noted in four patients, who experienced colitis (*n* = 3) and impaction colic (*n* = 1). The other 19 patients did not have any adverse effects during their stay in the hospital according to the medical records.

Follow-up was obtained from 11 cases in the meropenem group. Of these, seven reported that their horse was sound enough to return to normal activity, three to reduced activity, and one was retired completely. For the 10 horses that were able to return to work, it took an average of 9 +/– 4 months to return to work. Eight of these horses require treatments, such as anti-inflammatory (*n* = 4), steroid joint injections (*n* = 3), topical ointments (*n* = 2), and antibiotics (*n* = 1), as maintenance. One of these owners reported recurrence of the infection, four reported problems with wound healing and excessive scarring, and two reported arthritis related to the injury. After leaving the hospital two owners noticed the adverse effects of diarrhea (*n* = 2).

#### Gentamicin

Thirty-one of the 37 horses (84%) in the gentamicin group survived to discharge, but six were euthanized in hospital due to poor prognosis. The six horses that were euthanized prior to discharge were diagnosed with septic arthritis of the distal interphalangeal joint (*n* = 2), septic physitis and arthritis (*n* = 1), septic arthritis of the metatarsal-phalangeal joint (*n* = 1), septic arthritis and septic navicular bursa (*n* = 1), and septic arthritis and tenosynovitis (*n* = 1). The time to euthanasia was on average five days, ranging from one to 11 days. Adverse effects due to some aspect of the treatment regimen was noted in only five patients, who experienced colitis (*n* = 4) and impaction colic (*n* = 1). The other 32 patients did not have any adverse effects during their stay in the hospital according to the medical records.

Follow-up was obtained from 22 cases in the gentamicin group; of these, 12 reported that their horse was sound enough to return to normal activity, six to reduced activity, and four were retired completely. For the 18 horses that were able to return to work, it took an average of 7.69 +/– 4.46 months to return to work. Five of these horses require treatments, such as anti-inflammatories (*n* = 3), antibiotics (*n* = 1) and corrective shoeing (*n* = 1), as maintenance. Two of these owners reported recurrence of the infection, seven reported problems with wound healing and excessive scar tissue, and two reported arthritis related to the injury. After discharge from the hospital no owners noticed adverse effects.

#### Comparisons

The overall survival to discharge was 86% (52/60), with meropenem being 91% (21/23) and gentamicin being 84% (31/37). The differences in survival to discharge between groups were not statistically significant (*p* = 0.6983). When the outcomes of the horses that were contacted in each group were compared no statistically significant differences were detected between the meropenem and gentamicin groups with respect to return to work (meropenem: 7/11; gentamicin 12/22; *p* = 0.7193), reduced work (meropenem: 3/11; gentamicin: 6/22; *p* = 1) or retired/euthanized (meropenem: 1/11; gentamicin: 4/22; *p* = 0.6431). When horses with only positive culture results were compared within each group, no statistically significant differences in outcome were noted (Supplementary File 3).

## Discussion

Our study presents the clinical data and outcome for two groups of horses that had treatment augmented with IVRLP meropenem or gentamicin for diagnosis of orthopedic sepsis. While each clinical case is unique and workup can vary based on client resources, case presentation and clinician, the diagnoses were supported by either culture information, altered synovial fluid characteristics, diagnostic imaging, or a combination. Cytology data showed that the average cell counts and protein levels were well-above the normal value range reported to be <1,000 cell/μl (29). Infected synovial structures generally have over 30,000 cells/μl (28), which was seen in both groups. At a total protein over 3.0–4.0 g/dl the structure is likely infected (28, 29), which was the case for both groups in our study. While there were significant differences in the synovial fluid concentrations of both groups (meropenem: 5.42 g/dl; gentamicin: 4.35 g/dl; *p* = 0.0371) it is important to note that both groups had elevated total protein values which were consistent with infection (28).

Cases that had septic synovitis secondary to a penetrating wound was frequently the metacarpo/metatarsophalangeal joint which is consistent with other reports (11). According to previous studies, the most common infected joint due to trauma was the fetlock, which is similar to findings in this study (22). The majority of bacterial species isolated from cultures of meropenem treated horses were common bacterial species, similar to those found in other reports septic synovial structures (20). Positive cultures in this study also contained less commonly reported bacterial species in equine septic synovitis (e.g., *Actinobacillus equuli*), creating a diverse group. Trauma was the most commonly identified cause of infection for both gentamicin and meropenem groups, many of which were due to lacerations. The limbs that were affected were also similar in both groups, with the left hind limb having the most infections. Nine horses received meropenem as the first antibiotic given via IVRLP, meaning none of the other possible drugs were attempted first. For the 15 cases where other antibiotics were tried first, only three stated they switched due to results of culture. Almost half of the meropenem horses that had samples submitted for culture received the treatment before culture results were reported. At the institution represented in this study, protocols existed restricting the use of carbapenems to the rare use for severe, life threatening conditions in individual animals when culture and susceptibility testing indicates that it is the only suitable option, however, our results clearly indicate that in many of the reported cases, the necessary criteria for carbapenem use was not met. Retrospective analysis of antimicrobial use practices within the institution for adherence to this policy identified the frequent use of meropenem for IVRLP in horses and prompted the retrospective analysis presented here.

Of the 26 bacterial isolates obtained from culture in either group, 24 displayed MIC ≤1 μg/mL for imipenem and were reported by the laboratory as sensitive. In previous studies, when administered by IVRLP to healthy horses, the concentration of imipenem in the fetlock joint peaked at 60–87 μg/ml and was above 1 μg/ml for roughly 6 h at a 0.5 gm dose (16), suggesting that imipenem may be a viable antibiotic choice in most of these cases. While laboratory-reported susceptibilities to meropenem were not available, studies comparing treatment with meropenem to imipenem found no significant difference in the outcome of serious infections in humans (30). These two antibiotics also exhibit similar *in vitro* activity and pharmacokinetics (30). All carbapenems, including both meropenem and imipenem, typically produce MIC values of ≤ 1 μg/dl against the commonly isolated susceptible Gram-positive and Gram-negative bacteria in this study such as *Staphlyococcus* sp., *Streptococcus* sp., and *E. coli*. Therefore, extrapolation between imipenem testing, which is routinely tested for using commercially available antimicrobial susceptibility testing panels for equine patients in the United States, for clinical use of meropenem is reasonable (30), although future work is necessary to verify this extrapolation. While meropenem has been shown to have a variable synovial fluid concentration in healthy horses after IVRLP that may not remain above 1 μg/ml for longer than 3–4 h (19), that study used a 0.5 gm dose, and the horses in our study received 1 gram per treatment. While the pharmacokinetics of IVRLP are not as well-known as plasma pharmacokinetics of parenterally administered drugs, it is possible that with an increased IVRLP dosage, an increase of the maximum concentration could be expected, which could allow for higher concentrations to persist for an increased period of time compared to 0.5 gm dosing. Thus, 1 gm dosing may offer improved clinical efficacy over the 0.5 gm dosing that has previously been reported (19). However, laboratory-reported susceptibility to other antibiotics like ceftiofur (*n* = 22/26), ampicillin (*n* = 18/26), amikacin (*n* = 15/26), or gentamicin (*n* = 12/26) was also frequently present as seen in Table 4.

It is important to note that no clinical breakpoints exist specifically for IVRLP in horses and diagnostic laboratories report susceptible (S)/intermediate (I)/resistant (R) based on data related to systemic treatment with those antibiotics. Therefore, all of the interpretations (susceptible or resistant) that are reported for these cases are extrapolated from clinical breakpoints either for systemic use of the same drug for the same bacteria (only a few of these exist for horses) or, more likely, from breakpoints established in humans or other animal species for similar bacteria. In addition, there are no clinical breakpoints for meropenem or imipenem currently approved in any veterinary species, therefore, all interpretations for this class are extrapolated from human breakpoints. While some of these extrapolated breakpoints may closely align with the true epidemiologic cut-off value for non-wildtype (i.e., have acquired resistance genes) for the bacteria of interest, others may not and may be heavily influenced by the pharmacokinetics of the drug in a specific species. Therefore, the confidence level in these interpretations is unfortunately and represents a significant limitation to this study and to many other similar studies in veterinary medicine (31). The use of IVRLP, which leads to significantly higher concentrations at the site of infection when compared to systemic therapy, may be able to overcome increased MIC values which would be otherwise be classified as resistant in some cases. The antimicrobial susceptibility interpretations in this study were reported as the clinician would have seen them, based on what the lab reported, which may also vary from laboratory to laboratory based on which extrapolations are used in each case. In addition, in many of the cases IVRLP was performed with both an antibiotic as well as mepivacaine; while previous studies have shown no effect on antimicrobial activity of amikacin (an aminoglycoside similar to gentamicin) when combined with mepivacaine, no similar studies have been performed using meropenem (32). While the commonly isolated bacteria from equine synovial infections are typically susceptible to the mechanism of action of meropenem, as demonstrated in this study, resistance to meropenem is possible with strains of methicillin-resistant *Staphylococcus aureus* (MRSA) and carbapenemase-resistant Enterobacteriaceae (CRE) such as *Escherichia coli*, (33), therefore clinicians should also consider the efficacy of other antibiotics while making decisions on a IVRLP treatment plan (30).

Adverse effects were noted in four horses treated with meropenem but it cannot be determined if this was from concurrent clinical disease, this treatment, or from other medication given. The severe side effects seen in humans given meropenem, such as seizures (34), were not seen in this population. Additionally, the adverse effects of IVRLP of antibiotics described in other species and humans, such as venous scaring, transient pyrexia, thrombosis, bruising, and phlebitis were not observed or noted in the medical records examined in this study (25–27, 35). This lack of adverse effects are similar to what was previous found in a prospective study using healthy horses (19). The overall survival to discharge was 86% (52/60), with meropenem being 91% (21/23) and gentamicin being 84% (31/37). A previous study using various treatments in adult horses had an overall survival of discharge of 85%, very similar to both of the groups in this study (21). Due to the lack of owner response, outcomes were not determined for all horses enrolled in the study. Despite this, no significance differences were found between the outcome of the two groups, with both groups showing mostly positive outcomes. With these outcomes in mind, while meropenem may be an effective antibiotic for septic synovial structures, other more judicious antibiotic treatments may produce similar outcomes.

This study had several additional limitations. The number of horses receiving meropenem via IVRLP at the teaching hospital was limited so only a small population of horses were able to be enrolled in the meropenem group. The retrospective nature of this study is an additional limitation. There is also variability in the clinician treatment protocols, including IVRLP procedure, for the horses enrolled in the study. A variation in clinical presentation is also present, as horses with different stages of infection at different anatomical locations were compared. Not all cases had culture or cytology data so some were diagnosed with diagnostic imaging only, leading to an additional weakness. Variation in ancillary therapies is also a limitation. All of the horses enrolled in the study received systemic antibiotic treatment along with the IVRLP treatment, which limits the ability to identify the complete efficacy of the single agent in the specified treatment groups. Future studies could include multiple institutions, which would allow for more horses to be compared. Also, investigations evaluating specific bacterial diagnoses and treatment protocols would also help to reduce some of the variability for future work.

In conclusion, based on bacterial antimicrobial susceptibility testing and case outcomes, antimicrobial therapy augmented with IVRLP using either meropenem or gentamicin both appear to be an effective treatment for septic synovial structures. Although this study found treatment with meropenem may be effective, treatment with other antimicrobials of lesser critical importance in human medicine is the more ideal choice from an antimicrobial stewardship perspective. Bacterial species may be susceptible to other antibiotics, such as gentamicin, which showed similar outcomes in this study. Restricted antimicrobials, like meropenem, should only be used when the described guidelines, such as proven resistance to less restricted antimicrobials and/or a life-threatening case severe enough to outweigh the risks, are met. If another treatment can lead to a similar outcome, it is preferable option in order to help reduce the development of resistance to critically important antibiotics such as the carbapenems.

## Data Availability Statement

The original contributions presented in the study are included in the article/Supplementary Material, further inquiries can be directed to the corresponding author/s.

## Author Contributions

AM conceptualized the study, analyzed patient records as well as results, followed up with clients regarding outcomes, and constructed the manuscript. JS, DT, JT, and AK conceptualized the study, analyzed results, and constructed the manuscript. All authors contributed to and approved the final manuscript.

## Conflict of Interest

The authors declare that the research was conducted in the absence of any commercial or financial relationships that could be construed as a potential conflict of interest.
