# Supplementary material for: Meropenem Administered via Intravenous Regional Limb Perfusion for Orthopedic Sepsis in Horses: A Clinical Retrospective Study
Source: Front Vet Sci. 2021 Mar 26;8:629627. doi: 10.3389/fvets.2021.629627 (PMC8033006; doi:10.3389/fvets.2021.629627)
Supplement: Supplementary file 1 [file Data_Sheet_1.docx]

Outcome Sheet

Client name:

Horse name:

EMR#:

Month/year treated:

Diagnosis:

Survived to discharge? Y/N

Date called:

Answer? Y/N #

LMOM? Y/N #

Follow up?

Questions:

1. What was activity level and athletic discipline before injury/surgery/infection?
2. Return to full work (normal activity, same as before injury), limited/reduced work, trial horse retired (still lame)?
   1. If able to return to full work, about how long did it take to get to that point estimate in months?
3. Any type of treatments (special shoeing, long term meds/treatments, etc.) needed/following discharge to reduce/eliminate lameness currently?
4. Any recurrence of infection or severe lameness? Did the wound heal completely? Long term arthritis in the limb?
5. Did your horse experience any complications such as diarrhea, colic, wound healing (swelling, etc), or any other abnormalities after leaving the hospital?

Script for phone calls:

If answer:

Hi my name is ________ and I am a veterinary student at _______. I was wondering if you had about 2-4 minutes to answer some questions about your horse _____ (name)’s previous visit with our hospital.

Great! We have records that he/she was hospitalized for __________ in __________ (year). Start questions.
