# Supplementary material for: Meropenem Administered via Intravenous Regional Limb Perfusion for Orthopedic Sepsis in Horses: A Clinical Retrospective Study
Source: Front Vet Sci. 2021 Mar 26;8:629627. doi: 10.3389/fvets.2021.629627 (PMC8033006; doi:10.3389/fvets.2021.629627)
Supplement: Supplementary file 2 [file Data_Sheet_2.docx]

Supplemental File 2: Perfusate composition of horses administered meropenem or gentamicin via intravenous regional limb perfusion.

| Group | Perfusate Constituents | *n* |
| --- | --- | --- |
|  |  |  |
| Meropenem (*n* = 23) | Not stated | 17 |
|  | 10 ml mepivacaine, 30 ml saline | 2 |
|  | 3 ml mepivacaine, 12 ml saline | 1 |
|  | 60 ml saline | 1 |
|  | 15 ml mepivacaine, 15 ml saline | 1 |
|  | 13 ml mepivacaine, 20 ml saline | 1 |
|  |  |  |
| Gentamicin (*n* = 37) | Not state | 27 |
|  | 10 ml gentamicin, 10 ml mepivacaine | 2 |
|  | 10 ml gentamicin, 5 ml mepivacaine, 10 ml saline | 2 |
|  | 10 ml gentamicin, 15 ml mepivacaine | 1 |
|  | 10 ml gentamicin, 20 ml mepivacaine | 1 |
|  | 10 ml gentamicin, 50 ml mepivacaine | 1 |
|  | 10 ml gentamicin, 20 ml mepivacaine, 20 ml saline | 1 |
|  | 11 ml gentamicin, 10 ml mepivacaine, 39 ml saline | 1 |
|  | 20 mg morphine/10 ml gentamicin | 1 |
