# Supplementary material for: Meropenem Administered via Intravenous Regional Limb Perfusion for Orthopedic Sepsis in Horses: A Clinical Retrospective Study
Source: Front Vet Sci. 2021 Mar 26;8:629627. doi: 10.3389/fvets.2021.629627 (PMC8033006; doi:10.3389/fvets.2021.629627)
Supplement: Supplementary file 3 [file Data_Sheet_3.docx]

**Supplementary File 3.** Comparison of outcomes of horses administered Meropenem and Gentamicin via intravenous regional limb perfusion applied only to animals that had positive culture data reported (excluding imaging and cytology components of case definition). *P* < 0.05 considered statistically significant.

| Treatment | Survival to Discharge | Follow up | Returned to normal | Reduced work | Retired/No improvement  /Euthanized |
| --- | --- | --- | --- | --- | --- |
| Meropenem | 13/15 | 7 | 5/7 | 0/7 | 2/7 |
| Gentamicin | 10/13 | 8 | 6/8 | 1/8 | 1/8 |
| P = | 0.6389 |  | 1 | 1 | 0.5692 |

Follow up: Patients where follow up information was obtained prior to discharge.

Returned to normal: Patients that resumed activity similar to prior to their injury after discharge.

Reduced work: Patients that were able to return to normal activity, but at a lesser degree than prior to their injury.

Retired/No improvement/Euthanized: Patients that were retired from work, had no improvement observed, or were euthanized due to reduced capacity after their injury.
